# Impact of virtual reality distraction during colonoscopy vs intravenous deep sedation: Results of a single-center randomized controlled trial

**DOI:** 10.1055/a-2520-9768

**Published:** 2025-03-14

**Authors:** Anastasia Pavlidi, Lotfi Triki, Julien Mortier, Jacques Deviere, Arnaud Lemmers, Vincent Huberty, Patrice Forget, Mark Hannen, Caroline Quolin, Turgay Tuna, Daniel Blero, Marianna Arvanitakis

**Affiliations:** 1 504992 Department of Gastroenterology, Digestive Oncology and Hepatopancreatology, HUB Hôpital Erasme, Université Libre de Bruxelles, Brussels, Belgium; 2 504992 Department of Anesthesiology, HUB Hôpital Erasme, Université Libre de Bruxelles, Brussels, Belgium; 3 Anaesthesia, Institute of Applied Health Sciences, School of Medicine, Medical Sciences and Nutrition, University of Aberdeen, Department of Anaesthesia, NHS Grampian, Aberdeen, United Kingdom, Aberdeen, United Kingdom of Great Britain and Northern Ireland; 4 European Organisation for Research and Treatment in Cancer (EORTC), Brussels, Belgium; 5 Gastroenterology, CHR Namur, Brussels, Belgium; 6 Research Unit in Cardio-Respiratory Physiology and Exercise Nutrition, Faculty of Human Movement Sciences, Université libre de Bruxelles, Brussels, Belgium

**Keywords:** Endoscopy Lower GI Tract, CRC screening, Quality and logistical aspects, Sedation and monitoring, Performance and complications

## Abstract

**Background and study aims:**

Colonoscopy is associated with discomfort that requires intravenous sedation (IVS). The aim of this randomized controlled trial (RCT) was to explore the feasibility of virtual reality distraction (VRD) for colonoscopy using two primary endpoints: cecal intubation rate and the rate of rescue with IVS.

**Patients and methods:**

Patients scheduled for elective colonoscopy with IVS were randomized in a 2:1 ratio in favor of VRD, with rescue IVS by propofol if needed. VRD involved use of a device providing a visual and auditive experience similar to clinical hypnosis.

**Results:**

Ninety patients were included (VRD:60, IVS: 30). Cecal intubation rate was similar in both groups (92.8% for VRD vs 100% for IVS, *P* =0.3). The rate of rescue IVS in the VRD group was 63.6%. There was a decrease in median total dose of propofol per patient in the VRD group (1.15 mg/kg for VRD and 4.41 mg/kg for IVS, *P* <0.001) and in the subgroup of VRD patients who received IVS rescue (3.17 mg/kg for VRD and 4.41 mg/kg for IVS, *P* =0.003). The median level of pain was higher and the median level of comfort was lower in the VRD group (respectively 3 vs 0, *P* <0.001 and 7 vs 10, *P* <0.001).

**Conclusions:**

This RCT provides preliminary data to better understand the feasibility of VRD for colonoscopy. We have not identified differences in procedure outcomes compared with conventional IVS, but nevertheless, higher pain and lower comfort scores were reported.

## Introduction

1

Colonoscopy plays a crucial role in colorectal cancer (CRC) screening due to its high diagnostic sensitivity and therapeutic potential for treating premalignant colorectal lesions 1 2 . Guidelines from the American College of Gastroenterology recommend screening in average-risk individuals between the ages of 50 and 75 years and suggest screening in average-risk individuals between ages 45 and 49 years to reduce incidence of advanced adenoma, CRC, and CRC-related mortality 3 . Nevertheless, according to the U.S. Preventive Services Task Force (UPSTF), in 2016, 26% of eligible adults in the United States had never been screened for CRC and, in 2018, 31% were not up to date with screening 4 .

Colonoscopy is perceived as a painful and anxiety-generating experience associated with emotional discomfort and embarrassment, leading patients to be reluctant to undergo the procedure 5 . Reflex spasms caused by colonic stimulation and acute distention secondary to inflation are the main causes of pain during colonoscopy that require intravenous sedation (IVS) with propofol, either alone or in combination with benzodiazepines and narcotics 6 .

Although propofol sedation is largely used for endoscopic procedures, there is considerable variation in IVS practice between different centers and countries 7 8 . The main reason for this is the differing sociocultural backgrounds that influence the choices and attitudes of patients and endoscopists 9 .

Furthermore, use of sedation can be associated with adverse events (AEs), particularly in older patients 8 10 11 . Also, in some countries, propofol may only be administered by an anesthesiologist, resulting in potentially higher costs for the patient and the community 7 . Finally, IVS during colonoscopy can prolong recovery-room stay, delay discharge, and increase total medical costs 12 .

Therefore, it is imperative to find safe, easy-to-use, and cost-effective ways to relieve pain and anxiety for patients undergoing colonoscopy. Clinical hypnosis and virtual reality (VR) have demonstrated effects on pain and anxiety reduction when delivered during medical procedures 13 14 15 16 17 . Immersive VR technology can guide the patient through the same steps as those used when clinical hypnosis is induced through an interpersonal process 18 19 . However, efficacy of clinical use of VR distraction (VRD) during endoscopic procedures is still not well documented.

A VRD module, the Aqua module of the Oncomfort device (Oncomfort SA, Wavre, Belgium) was designed for management of pain and anxiety related to medical and surgical procedures. The purpose of this pilot study was to explore efficacy, safety, and pharmacoeconomic outcomes in patients undergoing colonoscopy with either VRD using the Aqua module or standard-of-care IVS with propofol.

### Patients and methods

#### Study center

This randomized controlled trial (RCT) was conducted at Erasme University Hospital in Brussels, Belgium, a teaching hospital associated with Université Libre de Brussels, between June 25, 2020 and December 21, 2021. The protocol was approved by the Ethics Committee of the study center (P2020/250) on the May 27, 2020 and was registered at ClinicalTrials.gov (NCT04465383). Written informed consent was obtained from all subjects before their inclusion in the study and the trial was performed in accordance with the principles of the Helsinki Declaration. This report was written in accordance with the CONSORT recommendations.

#### Study population

The study population included male and female adult patients (≥ 18 years old) with an indication for screening or diagnostic colonoscopy under conventional IVS (propofol), scheduled in the outpatient setting in the Day Clinic. Exclusion criteria can be found in Supplementary Table 1.

### Randomization

A prospective, randomized, controlled design was used. Participants were proposed for inclusion during the pre-procedure consultation with the anesthesiologist and, if they were willing to participate in the study, they were randomly assigned to one of two arms via computer-generated randomization concealed using opaque envelopes:

Group A- Experimental arm: VRD. Administration of rescue IVS (propofol) if needed upon patient request (VRD+IVS group).

Group B - Control arm: conventional IVS (propofol).

Random assignment to the study arms was done on a 2:1 basis (2:1 in favor of the experimental arm). This was decided upon to provide calibration of results observed in the control arm as well as to allow an increased sample size in the experimental arm, providing more information about VRD. Participants were informed of their group allocation directly before initiation of the colonoscopy procedure.

### DS protocol

The VRD session consisted of VR software (Aqua module Version 3.0 or subsequent version, Oncomfort SA, Wavre, Belgium) including a clinical hypnosis script. Patients in the intervention group underwent a 30-minute VRD program through the device.

Session duration could be adapted according to procedure duration through the device controller.

**Fig. 1:**
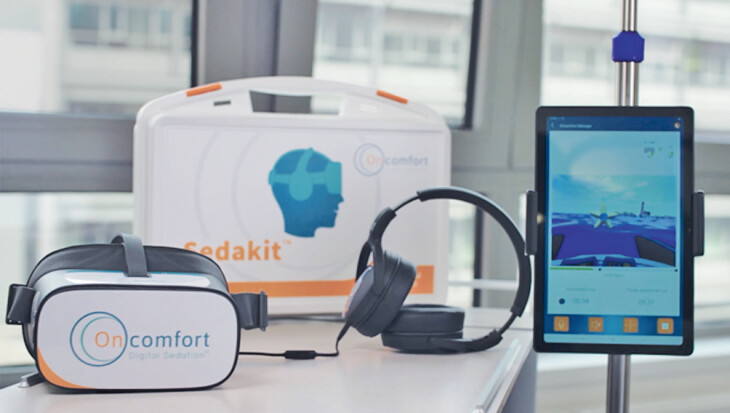
The Oncomfort device for virtual reality distraction.

For the VR protocol, the patient was asked to lay on their left side and the medical device was installed as a mask, projecting an underwater experience while playing a hypnotic script designed to induce a change in state of consciousness, increasing parasympathetic system tone and relaxation response, and reducing the perception of painful stimuli ( Fig. 1 ).

In case of patient complaint and at anesthesiologist discretion, rescue propofol sedation could be administered to the patient at any time (VRD + IVS). Rescue propofol sedation was administered to the patient in case of discomfort and/or patient complaint according to the score on the Behavioral Pain Scale (BPS). Patients with a BPS score > 3 were eligible for rescue propofol sedation. Propofol was administered with a target-controlled infusion, at a starting effect dose concentration (Ce) of 1 µg/mL to 6 µg/mL, adapted to weight (Marsh model) and incrementally increased by 0.5 µg/mL, if needed, depending on patient reaction. Practically, this corresponds to a starting dose of 20 to 30 mg at the beginning of procedure with additional doses of 10 to 20 mg administered during the procedure. Doses were adapted according to the level of comfort and needs of the patient. Recorded parameters throughout the procedure included peripheral oxygen saturation, mean arterial pressure (MAP), and continuous electrocardiogram.

### Conventional IVS protocol

The procedure was performed under propofol sedation, with a target-controlled infusion at a starting effect-site concentration (Ce) of 1 µg/mL to 6 µg/mL depending on patient reaction and incrementally increased (by 0.5 µg/ml). Doses were adapted according to the level of comfort and patient needs. This sedation corresponds to American Society of Anesthesiology level of sedation and analgesia of 3 to 4 (moderate/conscious to deep sedation/analgesia). Use of other drugs, such as midazolam, ketamine, or fentanyl, was not prohibited but was avoided as much as possible. Recorded parameters throughout the procedure were no different than in the VRD group. Patient discharge from the recovery room was based on the Aldrete Score System (≥9/10) 20 .

### Study endpoints

In the first stage, the primary objective of this study was to evaluate the feasibility of using VRD in colonoscopy. To facilitate this evaluation focusing on two primary endpoints, cecal intubation rate and rate of rescue sedation requirement in the VRD group were used. Secondary endpoints are listed in Table 1 .

**Table:** **Table 1** Secondary endpoints.

1	To compare use of sedation medication in both arms: Average (per patient) total doses of sedation medication per arm
2	To compare performance of colonoscopy in both arms: Adenoma detection rates, total duration of colonoscopy, total duration of sedation, total length of stay in the procedure room, total length of stay in the recovery room, total length of stay in the hospital
3	To assess difficulty or ease of the procedure in both arms: Number of abdominal compressions and need for patient position changes
4	To compare patient experience in both arms: Pain by Visual Analogue Scale (VAS 0 to 10), anxiety by Visual Analogue Scale (VAS 0 to 10), comfort by Visual Analogue Scale (VAS 0 to 10), preferred type of sedation, recall of the procedure, willingness to repeat the same procedure, AE rates
5	To assess gastroenterologist and anesthesiologist satisfaction in both arms: Measure of satisfaction of perceived facilitation or impediment of the procedure by digital sedation as measured by Likert scale (0 to 5)
6	To assess gastroenterologist and anesthesiologist stress in both arms: Measure of stress by Visual Analogue Scale (VAS 0 to 10)
7	To compare physiologic measures in both arms: Oxygen saturation (minimal observed during procedure) and MBP (minimal/maximal values)
AE, adverse event; MBP, mean blood pressure; VAS, Visual Analogue Scale.	

### Sample size

Study sample size was computed based on expectations for the experimental arm at final analysis. Randomization (2:1 in favor of the experimental arm) was the aim mostly to provide calibration of the results observed in the control arm. However, hypothesis-testing was planned for selected secondary efficacy endpoints, even though no power calculations had been done for these tests. In order to demonstrate that the cecal intubation rate in the experimental arm was ≥90%, 107 evaluable patients would be needed in the experimental ar, in order for the one-sided 95% exact confidence interval for the observed cecal intubation rate to be entirely higher than 90% (considering an expected observed rate of 95%, or 102/107 patients with cecal intubation). Assuming loss of data or follow-up in the experimental arm of 10%, a total of 118 patients needed to be randomized to the experimental arm. Given the 2:1 randomization, the initial calculated total number of patients needed for the study was 177. Both co-primary endpoints were considered to assess study positivity; for this reason, no multiple-testing corrections were needed for calculation of confidence intervals (Cis) and sample sizes.

### Interim analysis

An interim analysis was planned when 25% of the evaluable patients had been recruited to the experimental arm (27 evaluable patients). If 21 or fewer of the 27 patients had successfully undergone cecal intubation (i.e., a cecal intubation rate of 78%), the probability that the true rate would have been at least 90% was below 5%, and, therefore, continuation of the trial would be deemed futile. Likewise, if 19 or more of the 27 evaluable patients had received rescue sedation (a rate of 70%), the probability that the true rate would have been at most 50% was below 5%, and futility could be declared. Both futility boundaries were non-binding, which meant that definitive decisions about stopping the trial were left to investigator discretion and had no formal regulatory implications.

### Analysis sequence

Interim analysis and final analysis of the co-primary endpoints were conducted in the per-protocol population, defined as patients who did not request rescue IVS before introduction of colonoscopy (in the experimental arm) and did not withdraw their consent (in both arms).

Thereafter, the study was initially divided into three stages, with randomization (2:1) maintained throughout. In the first stage, accrual was halted when about 45 patients had been randomized, at least 27 of whom being evaluable patients randomized to the experimental arm. A non-binding futility analysis was planned for the primary outcome. In the second stage, accrual was halted when about 90 patients had been randomized. A non-binding futility analysis was planned for the primary outcome. At the end of recruitment of Stage 1 and 2, an analysis of the secondary outcomes would also be made. Data obtained until that stage were consistent with statistical power. Adding to this, the study took place during the pandemic, which complicated recruitment and organization of the study. Thereafter, the study did not continue to the planned final accrual of 177 patients (118 to experimental and 59 to control arm, respectively) and recruitment was completed at Phase 2.

### Statistical analysis

The analysis was applied using R-studio software. This consisted of a per-protocol analysis with no replacement of missing data. Qualitative data are described by frequencies and percentages. Normally distributed quantitative data are described by means and standard deviations. We estimated the probability density of the data using violin plots to see if our data were normally distributed. Non-normally distributed data are described by the median, first, and third quartile values. Chi-squared tests were used to compare frequencies and rates between groups. In cases where the assumptions of the chi-squared test were violated, particularly when the expected values were <5, Fisher’s exact test was used as an alternative. A Student’s *t* -test was used to determine whether there were any significant differences between the means of normally distributed data between the two groups. Alternatively, the Mann-Whitney-Wilcoxon test was used to compare asymmetrical distributions. The level of significance was 5% throughout all analysis.

## Results

**Fig. 2:**
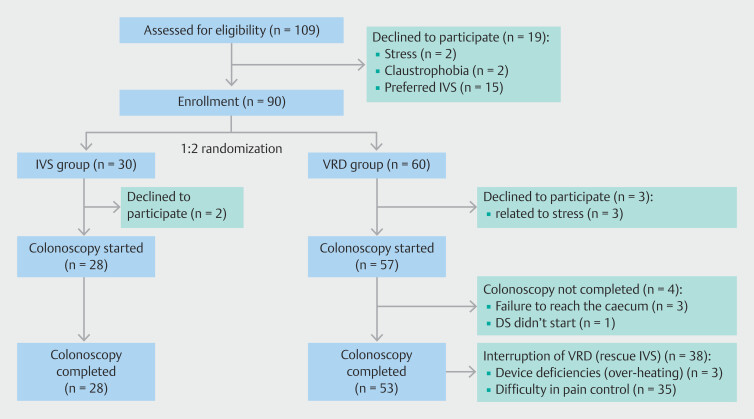
Study flowchart. VRD, virtual reality distraction; IVS, intravenous sedation.

A total of 109 patients were initially screened and were consented to the study, of whom 19 were excluded (withdrawal because of stress, claustrophobia, and preference for IVS). Consequently, a total of 90 patients entered the study and were randomized between the two colonoscopy arms on a 2:1 basis: 60 in the VRD group, 30 in the IVS propofol group ( Fig. 2 ).

Five patients did not undergo colonoscopy and were excluded. Thus, a total of 85 patients underwent colonoscopy (28 IVS, 57 VRD) and data for these procedures were entered into the final statistical analyses. No significant differences were observed between the groups regarding demographic factors. Demographic details of the two groups are presented in Table 2 .

**Table:** **Table 2** Demographic characteristics of the study population.

Variable	VRD group (n=57)	IVS group (n=28)	Total (n=85)
Age, yr, mean (SD)	55.4 (13.5)	55.7 (16.3)	55.5 (14.4)
Male sex, no. (%)	23 (40.4)	17 (60.7)	40 (47.1)
Height, cm, mean (SD)	168.2 (8.7)	172.8 (9.3)	169.8 (9.1)
Weight, kg, mean (SD)	75.3 (13.7)	74.4 (14.1)	75.0 (13.8)
BMI, mean (SD)	26.6 (4.9)	24.8 (4.0)	26.1 (4.7)
Presence of significant medical comorbidities, no. (%)	40 (70.1)	16 (57.1)	56 (65.9)
History of sleep apnea, no. (%)	8 (14.0)	2 (7.1)	10 (11.8)
Previous colonoscopy, no. (%)	39 (68.4)	20 (71.4)	59 (69.4)
BMI, body mass index; IVS, intravenous sedation; SD, standard deviation; VRD, virtual reality distraction.			

### Primary and secondary endpoints

Results for primary and secondary endpoints are shown in Table 3 . Cecal intubation rates were 92.8% in the VRD group vs 100% in the IVS group ( *P* =0.365). The rescue sedation rate was 63.6% in the VRD group (38/60 patients). There was a reduction in median total dose of propofol (mg/kg) per patient: 73.9% reduction in the VRD group as compared with the IVS group (1.15 vs 4.41 mg/kg, *P* <0.001) and in the subgroup of VRD patients who received IVS rescue (3.17 mg/kg for DS and 4.41 mg/kg for IVS, *P* =0.003), as well as shorter median duration of sedation in the VRD group (6 vs 20.5 [min], *P* <0.001).

**Table:** **Table 3** Results of primary and secondary endpoints.

Variable	VRD Group	IVS Group	*P* value
Primary endpoints			
Cecal intubation rate, no./total no. (%)	53/57 (92.8)	28/28 (100)	0.365
Rate of rescue sedation, no./total no. (%)	38/60 (63.6)	–	–
**Secondary endpoint: Amount of sedation**			
Total dose of sedation (mg/kg) per patient, median (IQR)	1.15 (0–4.01)	4.41 (3.58–6.83)	<0.001
Total duration of sedation in min, median (IQR)	6 (0–19.5)	20.5 (15.25–29.75)	<0.001
**Secondary endpoint: Colonoscopy performance measures**			
Adenoma detection rate, %	44.6	46.4	1
Biopsies taken, %	60.0	60.7	1
Need to change position rate, %	19.6	10.7	0.367
Need for abdominal compressions, %	75	53	0.367
Total procedure duration in min, median (IQR)	22 (16.7–34)	20.5 (15–28.7)	0.599
**Secondary endpoint: Hospital flow**			
Total length of stay in procedure room in min, median (IQR)	46.5 (40.7–58.5)	41.5 (35–50.5)	0.031
Total length of stay in recovery room in min, median (SD)	39.5 (21.4)	54 (20.2)	0.020
Total length of hospital stay in min, median (IQR)	200 (139–259)	184 (150–216)	0.500
**Secondary endpoint: Patient experience during procedure**			
Anxiety, VAS scale Median (IQR) (0=not at all anxious, 10=extremely anxious)	3 (1–5)	2 (1–3.25)	0.115
Comfort, VAS scale, Median (IQR) (0=not at all comfortable, 10=extremely comfortable)	7 (5–8)	10 (9–10)	<0.001
Pain, VAS scale, Median (IQR) (0=not at all painful, 10=extremely painful)	3 (2–5)	0 (0–0)	<0.0001
Capacity of recalling the procedure, Likert scale, Median (IQR) (0=negative memory of the experience, 5=positive memory)	4 (3–5)	5 (4.7–5)	<0.0001
AEs (number)	2: weakness, hands tremor	2: bradycardia, hypotension	
**Secondary endpoint: HCP experience**			
Evaluation of anesthesiologist, Likert Scale, Median (IQR) (0=not at all satisfied, 5=totally satisfied)	3 (2–5)	5 (5–5)	<0.001
Evaluation of gastroenterologist, Likert Scale, Median (IQR) (0=not at all satisfied, 5=totally satisfied)	4 (3.7–5)	5 (5–5)	0.010
Level of stress of anesthesiologist, VAS scale, Median (IQR) (0=not at all stressed, 10=totally stressed)	3 (1–5)	0 (0–1)	<0.001
Level of stress of gastroenterologist, VAS scale, Median (IQR) (0=not at all stressed, 10=totally stressed)	2 (0–3)	0.5 (0–1)	<0.001
**Secondary endpoint: Physiologic measures**			
Oxygen saturation, % (median minimal value observed during procedure, IQR)	97 (95–99)	97 (96–99)	0.736
MAP, mmHg (median minimal and maximal value observed during procedure, IQR)	Minimal value=87 (75–103) Maximal value=111 (96–123)	Minimal value=75 (68–82) Maximal value=97 (83–113)	<0.001 0.003
Heart rate, beats per minute (mean minimal and maximal value observed during procedure, SD)	Minimal value=66.7 (12.2) Maximal value=86 (14.2)	Minimal value=61.7 (10.3) Maximal value=77 (13.1)	0.079 0.010
AE, adverse event; HCP, healthcare professional; IQR, Interquartile range; IVS, intravenous sedation; MAP, mean arterial pressure; Q1-Q3 values; SD, standard deviation; VAS, visual analogue scale; VRD, virtual reality distraction.			

There was no difference between the groups regarding other colonoscopy parameters such as adenoma detection rate, biopsies performed, need to change position, and procedure duration. A higher proportion of patients required abdominal compressions during colonoscopy in the VRD group compared with the IVS group (75% vs 53%, *P* =0.367). Concerning the impact on hospital flow, patients in the VRD arm stayed longer in the procedure room than those in the IVS arm (median time 46.5 vs 41.5 [min], *P* =0.031) but stayed for a shorter time in the recovery room (median time 39.5 vs 54 [min], *P* =0.020) with no difference in total duration of stay in the hospital.

Reported median level of pain during colonoscopy was higher in the VRD group as compared with the IVS group (3 vs 0, *P* <0.001) and median level of comfort during colonoscopy was lower in the VRD group (7 vs 10, *P* <0.001) ( Table 3 ).

Regarding healthcare professional (HCP) experience, there was an overall lower satisfaction level for both anesthesiologists and endoscopists in the VRD group, as well as a higher level of stress. Anesthesiologist evaluations were correlated with patient pain (r=-0.44, *P* =0.017) and gastroenterologist evaluations were correlated with number of manipulations (r=-0.35, *P* =0.04).

Regarding physiological measures, there was no difference between the groups regarding oxygen saturation. There was a higher MAP in the VRD group compared with the IVS group.

Four AEs occurred in four different patients, two in the IVS group (bradycardia, hypotension) and two in the VRD group (tiredness, hand tremors).

## Discussion

This RCT is the first, to our knowledge, to investigate feasibility of colonoscopy with VRD without showing differences in terms of rate of cecal intubation compared with standard IVS with propofol. Even though more than half of the patients in VRD arm needed propofol rescue IVS, we found that the total dose of propofol was lower compared with the IVS arm, suggesting that pharmacological sedation can be minimized or even eliminated for some patients. Nevertheless, it must be noted that reported pain levels were higher in the VDR group, thus suggesting that the best alternative method to sedation is still to be determined, especially regarding the diversity of patient experience.

One of the advantages of VRD is reduction in hemodynamic AEs such as arterial hypotension, which requires increased hemodynamic monitoring and a longer stay in the recovery room. In a retrospective study, it was reported that sedation with propofol could lead to up to 30% rates of arterial hypotension 21 . Compared withthis study, there was no occurrence of arterial hypotension in the VRD group, even with rescue IVS, whereas there was one patient in the IVS group who presented with hypotension.

Visual distraction during colonoscopy is an effective way for improving patient pain, with higher rates of willingness to repeat the procedure, suggesting that audiovisual diversion techniques, such as VR, can improve patient acceptance and satisfaction of colonoscopy 6 12 22 23 24 . Table 4 summarizes results of all studies that have been conducted using VR during gastrointestinal endoscopy procedures in adult populations. Three previous RCTs comparing colonoscopy without sedation and colonoscopy with some type of VRD observed lower pain and anxiety scores in the intervention groups 25 26 27 . A small RCT compared colonoscopy under conscious sedation (midazolam) with colonoscopy with VRD, showing comparable performance measures (duration and completion of colonoscopy) 23 . Finally, a recent cohort study assessing use of VR in patients undergoing colonoscopy without sedation showed that 96.3% completed their colonoscopy without requesting or receiving any sedative medication 24 28 .

**Table:** **Table 4** Results of studies using VR during gastrointestinal endoscopic procedures in adult populations.

Author (year)	Country	No. of patients (Intervention group/control group if applicable) and study type	Procedure type	Device used	Primary outcomes
Lee (2004) 29	China	52/53 RCT	Colonoscopy under propofol	Eyetrek system with earphones (Olympus,Japan)	Significantly lower dose of propofol in intervention group (0.81 mg/kg +/0.49 vs 1.18 mg/kg +/-0.60) ( *P* <0.01).
Umezawa (2015) 26	Japan	28/29 RCT	Colonoscopy without sedation	Head-mounted display (Moverio Epson; Seiko Epson Corporation, Japan)	No significant difference in anxiety score between groups (median scores, 20 vs 24). Lower pain score in intervention group but not significantly different (median scores, 24.5 vs 42). Significantly higher median post-procedure satisfaction levels (median scores, 89 vs 72, *P* = 0.04).
Veldhuijzen (2020) 23	Netherlands	10/9 RCT	Colonoscopy under conscious sedation with midazolam and/or alfentanyl	Samsung Gear VR (Consumer Edition– SM-R322, combined with Galaxy S7, Korea)	Comparable values concerning: time to reach the cecum (median 10.48 minutes in the control group, versus 6.83 minutes in the intervention group) time to complete procedure (median 21.20 minutes vs 22.60 minutes) completed colonoscopies (100 % versus 90%) initial intravenous bolus of sedatives and analgesics, i.e., dose of midazolam (median, 2.5 mg in both groups), dose of alfentanyl (median, 0.25 mg in both groups).
Karaveli Çakır (2021) 27	Turkey	30/30 RCT	Colonoscopy without sedation	Cardboard Super Flex Goggles	No significant difference between pre- and post-operative state anxiety score between both groups but significant difference found between anxiety scores ( *P* <0.000) and pain scores ( *P* <0.03) during the procedure in favor of the intervention group.
Friedman (2021) 24	USA	27 Cohort prospective study	Colonoscopy with pharmacological rescue if needed	Samsung (Ridgefield Park, New Jersey, United States) Gear VR Oculus headset	96.3% (26/27) completed their colonoscopy without requesting or receiving any sedative medication.
Liu (2022) 25	China	58/59 RCT	Colonoscopy without sedation	Head-mounted VR display (Nibiru 3.50.005, Nanjing, China) and wristband (Empatica, Milan, Italy)	The median (IQR) pain scores were 7 (6–8) and 5 (4–6) in the control and intervention groups, respectively ( *P* <0.001).
Boonreunya (2022) 30	Thailand	32/32 RCT	Upper gastrointestinal endoscopy with topical anesthesia	Oculus GO (Standalone VR headset)	No statistical difference in pain during esophageal intubation between the groups (2.7±2.4 in the intervention group vs 2.3±2.3, *P* =0.751).
IQR, interquartile range; RCT, randomized controlled trial; VR, virtual reality.					

In contrast to these other studies, this study showed that the level of pain during colonoscopy was significantly higher in the VRD group compared with the IVS group but the level of pain after the procedure was similar between the two groups. There could be several explanations for why VRD did not significantly reduce the level of pain. First, this could be correlated with patient anxiety due to their concern about the VRD procedure, which is a first-time experience and probably perceived as having a limited antalgic effect due to lack of public awareness about this type of alternative modality 31 32 . Perin et al. demonstrated that an immersive three-dimensional (3D)-supported informed consent improved patient comprehension of their condition without increasing anxiety 32 . Therefore, such standard pre-procedure 3D reconstructions could be applied in our protocol, offering an immersive informed consent procedure prior to undergoing colonoscopy, and thus, acting as a psychological form of reassurance regarding the procedure 29 33 . Another hypothesis that could explain the higher levels of pain observed in this study is the increased number of abdominal compressions needed during colonoscopy in the VRD group. This could provoke a more painful and less comfortable experience for patients, as well as distracting them from the VR experience and decreasing its effect. In addition, this could be correlated with the higher level of stress in the HCPs, both gastroenterologists and anesthesiologists, because patients are conscious during the procedure and can still interact with the HCP team. The current study could not identify any factors predictive of response to VRD due to the small sample size. Finally, there may be discrepancies between reported level of pain (depending on consciousness level) and patient experience.

Compared with sedation modalities, VRD has potential advantages. First, VRD is standardized and does not depend on the skill or availability of the clinician compared with IVS, which needs a trained HCP who is responsible for maintenance of sedation and patient monitoring while using IVS. This could eliminate the need for clinician physical presence and increase capacity for reaching a greater number of patients who could benefit from VRD. Also, this could be useful for patients in rural areas and underserved regions 24 30 . In addition, without IVS, patients can return to work and drive almost immediately post-procedure, improving patient satisfaction levels. VRD during colonoscopy could also reduce health care costs compared with use of IVS, especially when an anesthesiologist is required.

Comparatively, VRD equipment is a one-time buy and dedicated software subscriptions are becoming more affordable with increasing use of the technology 31 . Another potential benefit of VRD is its lower environmental impact because it consists of a reusable machine with which a significant reduction in total dose of propofol was needed compared with IVS. Interestingly, it has been shown that propofol represents 45% of all drug-related waste in hospitals because the remaining propofol after each use cannot be stored 30 . Finally, deployment of non-anesthesia sedation is crucial after consideration of risks of over-sedation and related AEs. Consequently, VRD could be more appropriately used in patients who are at increased risk for AEs during IVS. High-risk factors for AEs during endoscopy procedures, such as male sex, ASA class of 3 or higher, and increased body mass index 10 , could be used for early identification of these complex cases for whom DS could be proposed.

### Limitations

This study has several weaknesses that should be taken into consideration when interpreting the results. First, the small sample size, as well as the fact that it was a monocentric study, means that there is a possibility that our results may not be reproducible and the study power may be overestimated, especially regarding cecal intubation rate. In addition, other quantitative parameters such as time to cecal intubation were not reported. Second, this study had some real-life issues because of the pandemic with difficulties in recruitment and organization. Third, all gastroenterologists involved in the study were trained to perform their examinations with IVS, which could lead to involuntary painful scope handling. Moreover, the study took place in an academic hospital with a high total number of HCPs participating, each one with a different background. A fourth limitation is the selection process for the rescue sedation group. Indeed, the BPS score was not strictly respected and applied; therefore, the selection process was not solely based on objective criteria, but mostly on anesthesiologist professional experience and confidence. Moreover, use of additional drugs during anesthesia was not reported. A fifth limitation is that the anesthesiologists were not blinded to patient assignment and could potentially introduce bias to outcomes.

## Conclusions

In conclusion, this was the first RCT to explore VRD, with optional IVS when needed, for colonoscopy. Despite similar procedure outcomes compared with conventional IVS, VRD has a less favorable pain and comfort profile. Furthermore, the group of patients who could benefit the most from non-sedating alternative modalities is still to be defined. Further development of alternative non-sedating modalities that provide satisfactory management of colonoscopy-related pain and discomfort while ensuring high-quality procedures could help increase rates of CRC screening.

## Footnotes

* Shared last authorship.

## Supplementary Material

Supplementary Material Zusatzmaterial
