# Supplementary material for: Impact of virtual reality distraction during colonoscopy vs intravenous deep sedation: Results of a single-center randomized controlled trial
Source: Endosc Int Open. 2025 Mar 14;13:a25209768. doi: 10.1055/a-2520-9768 (PMC11922172; doi:10.1055/a-2520-9768)
Supplement: Supplementary file 1 — Supplementary Material [file 10-1055-a-2520-9768_25266258.pdf]

Supplementary Table 1 Exclusion criteria.

1. Scheduled therapeutic procedure (resection, dilation)
2. Active Crohn’s disease
3. Low auditory acuity that precludes use of the device
4. Low visual acuity that precludes use of the device
5. Head or face wounds precluding use of the device
6. Schizophrenia
7. Dizziness
8. Water/sea phobia
9. Non-proficiency in French or Dutch (research language)
10. Factors precluding examination taking place in the Day clinic setting, such as important co-morbidity factors

Endosc Int Open | DOI 10.1055/a-2520-9768 | © The Author(s).
